# Trauma History and Depression Predict Incomplete Adherence to Antiretroviral Therapies in a Low Income Country

**DOI:** 10.1371/journal.pone.0074771

**Published:** 2013-10-04

**Authors:** Kathryn Whetten, Kristen Shirey, Brian Wells Pence, Jia Yao, Nathan Thielman, Rachel Whetten, Julie Adams, Bernard Agala, Jan Ostermann, Karen O'Donnell, Amy Hobbie, Venance Maro, Dafrosa Itemba, Elizabeth Reddy, for the CHAT Research Team

**Affiliations:** 1 Center for Health Policy, Duke Global Health Institute, Duke University, Durham, North Carolina, United States of America; 2 Duke Global Health Institute, Duke University, Durham, North Carolina, United States of America; 3 Department of Medicine, Division of Infectious Diseases and International Health, Duke University, Durham, North Carolina, United States of America; 4 Department of Epidemiology, The University of North Carolina at Chapel Hill, Chapel Hill, North Carolina, United States of America; 5 Department of Psychiatry and Behavioral Sciences, Duke University, Durham, North Carolina, United States of America; 6 Center for Child & Family Health, Duke University, Durham, North Carolina, United States of America; 7 Kilimanjaro Christian Medical Centre, Moshi, Tanzania; 8 Tanzania Women's Research Foundation, Moshi, Tanzania; Johns Hopkins Bloomberg School of Public Health, United States of America

## Abstract

**Background:**

As antiretroviral therapy (ART) for HIV becomes increasingly available in low and middle income countries (LMICs), understanding reasons for lack of adherence is critical to stemming the tide of infections and improving health. Understanding the effect of psychosocial experiences and mental health symptomatology on ART adherence can help maximize the benefit of expanded ART programs by indicating types of services, which could be offered in combination with HIV care.

**Methodology:**

The Coping with HIV/AIDS in Tanzania (CHAT) study is a longitudinal cohort study in the Kilimanjaro Region that included randomly selected HIV-infected (HIV+) participants from two local hospital-based HIV clinics and four free-standing voluntary HIV counselling and testing sites. Baseline data were collected in 2008 and 2009; this paper used data from 36 month follow-up interviews (N = 468). Regression analyses were used to predict factors associated with incomplete self-reported adherence to ART.

**Results:**

Incomplete ART adherence was significantly more likely to be reported amongst participants who experienced a greater number of childhood traumatic events: sexual abuse prior to puberty and the death in childhood of an immediate family member not from suicide or homicide were significantly more likely in the non-adherent group and other negative childhood events trended toward being more likely. Those with incomplete adherence had higher depressive symptom severity and post-traumatic stress disorder (PTSD). In multivariable analyses, childhood trauma, depression, and financial sacrifice remained associated with incomplete adherence.

**Discussion:**

This is the first study to examine the effect of childhood trauma, depression and PTSD on HIV medication adherence in a low income country facing a significant burden of HIV. Allocating spending on HIV/AIDS toward integrating mental health services with HIV care is essential to the creation of systems that enhance medication adherence and maximize the potential of expanded antiretroviral access to improve health and reduce new infections.

## Introduction

Globally, an estimated 33.4 million people are living with HIV infection, with over two-thirds living in sub-Saharan Africa (SSA). With the allocation of donor resources through the Global Fund to Fight AIDS, Tuberculosis, and Malaria, the US President's Emergency Plan for AIDS Relief (PEPFAR), and the World Bank Multi-Country AIDS Program, the number of people receiving antiretroviral therapy (ART) in less wealthy nations has increased from roughly 400,000 in 2003 to more than 8 million in 2012 [1]. In Tanzania, about 370,000 people, or 20% of the estimated 1.4 million people eligible for ART, are receiving treatment [2]. Recent guidelines, which recommend earlier initiation of ART at a CD4 cell count of 350 cells/mm^3^, are expected to rapidly increase the number of persons on ART over the next several years. The recent Institute of Medicine (IOM) evaluation of PEPFAR, while lauding many successes of the program, identified as an essential need the focus on improved medication adherence among patients [3].

Crucial to the success of ART is strict adherence to prescribed medication regimens. Incomplete adherence leads to drug resistance [4], [5] immunologic decline [6], faster progression of disease [7], and death [8]. Incomplete adherence has been a major challenge in HIV treatment worldwide, with adherence in the US estimated at 55% and, in SSA approximately 77% [9]. Higher adherence rates in SSA have dispelled doubts that ART would not be feasible in such resource-constrained settings and have pointed to the importance of tight social relationships in mediating adherence [10]. However, longitudinal data are sparse, and existing adherence studies are primarily among relatively new initiates of ART [7], [8]. Longer time on medication has been correlated with declining adherence in SSA [11]. In the Kilimanjaro region, Ramadhani et al. demonstrated that despite relatively high rates of adherence among patients who had been receiving ART for 6 months or longer, lack of complete virologic suppression was identified in 32% and ART resistance was identified in 10% of 150 patients [12].

In wealthier nations, psychosocial characteristics including having experienced past potentially traumatic events, life events, depression, anxiety, stress, beliefs about the efficacy of therapy, and patient-provider relationships are highly correlated with adherence [12], [13], [14]. Our own work has highlighted the importance of past traumatic events, depression and anxiety on ART adherence in the United States [15]. A recent qualitative study, in which 61 Tanzanian adults prescribed ART were interviewed about determinants of adherence found that psychosocial characteristics including religious beliefs, support from family and friends, and stigma influenced patients' beliefs and motivation about ART adherence [16]. Baseline findings from the CHAT study found high rates of exposure to potentially traumatic events [17].

This study examines associations among stressful and potentially traumatic events, current depressive symptoms and post-traumatic stress symptomatology, resource constraints as measured by having to make trade-offs between health care and other needs, social support, demographics, and adherence to ART among HIV+ clients in care at two regional hospitals and in four voluntary HIV counselling and testing sites. While adherence to ARTs in less wealthy nations is possible and high rates can be achieved, the authors hypothesize that, as in wealthy nations, the experience of potentially traumatic events and the mental health symptomatology of depression and anxiety will be associated with higher likelihood of incomplete adherence over time.

## Methods

This study and all study activities were approved by the Institutional Review Boards at Duke University in Durham, North Carolina, USA, the Kilimanjaro Christian Medical College and the National Medical Research Institute in Tanzania, and written consent was obtained from all participants.

CHAT is a longitudinal cohort study with the primary aim of understanding if, and to what extent, self-reported HIV medication adherence in a low income country is related to psychosocial factors such as potentially traumatic experiences, depression, post-traumatic stress symptomatology, and social support, as well as other socioeconomic and demographic factors. CHAT participants were enrolled in the study beginning in November 2008. Enrolment included 499 randomly selected HIV+ persons from the two local hospital-based HIV clinics and 267 newly diagnosed HIV+ persons randomly selected from four free-standing voluntary HIV counselling and testing (VCT) sites. These analyses included those interviewed at 36 months: 438 from the HIV clinics and 183 newly diagnosed HIV+ at baseline. Of those, 364 participants from the hospital-based clinics were taking ART and completed questions needed for this analysis, as were 104 from four free-standing voluntary HIV counselling and testing sites and completed questions needed for this analysis. Three of the latter tested HIV- at baseline, but seroconverted and started taking medication prior to the 36-month interview.

Participants completed detailed interviews with trained, field-certified interviewers at six-month intervals. The 36-month survey was used in these analyses because it included the most extensive measurements relevant to the research question.

## Measures

### Potentially traumatic events

The number of types of lifetime potentially traumatic events is a widely used trauma assessment that has been associated with multiple negative health outcomes [18], [19], [20], [21], [22]. The Life Events Checklist is used in conjunction with the system checklist described below to screen for and diagnosis anxiety and PTSD. The assessment measured 15 types of experiences including sexual and physical abuse, childhood physical and emotional neglect, and 11 other potentially traumatic childhood and adulthood experiences [23], [24], [25], [26]. The score ranges from 0 to 15, reflecting the number of types of potentially traumatic events experienced in the participant's lifetime.

Sexual abuse is defined in the scales to include sexual experiences (e.g., touching, intercourse) where force or threat of force was used; additionally, in children before the age of puberty the threat of force or harm was implied by a 5-year age difference between victim and perpetrator. Physical abuse was defined as incidents separate from sexual abuse in that they (1) were perceived to be life threatening (being physically attacked with the intent to kill or seriously injure), or (2) identified as other physical attacks in which victims were being beaten, hit, kicked, bit, or burned. Childhood physical and emotional neglect were measured with the Childhood Trauma Questionnaire and scored using cut-offs suggested by Bernstein and Fink for moderate physical neglect (≥9) and moderate emotional neglect (≥12) [26]. Additional childhood trauma assessments included the following events prior to age 18: (1) parental alcohol/drug abuse, (2) parental mental illness or parental suicide/attempted suicide, (3) imprisonment of a parent, (4) domestic violence/threat in the home, (5) child was placed in reform school/foster care/orphanage or was put up for adoption, (6) imprisonment of child, (7) homicide of a close family member/friend, (8) death of an immediate family member not due to homicide or suicide, and (9) child suffered from a life-threatening illness/injury not related to HIV. Additional potentially traumatic events assessed from adulthood included (1) homicide of a close family member/friend, (2) non-homicidal death of a child, and (3) non-homicidal death of a spouse/partner.

### Post-traumatic stress disorder symptoms

Post-traumatic stress disorder (PTSD) symptom severity was assessed with the PTSD Symptoms Checklist (PCL) based on DSM-IV criteria that include re-experiencing a traumatic event, numbing/avoiding, and hyperarousal symptoms. The scale is scored on a range of 0–68 with higher scores indicating greater PTSD symptomatology; it has strong reliability and correlation with a clinician-administered PTSD measure [27], [28].

### Depression

Depressive severity was measured using the Patient Health Questionnaire (PHQ) -9, a depression case identification tool that has been widely used and validated, including in SSA. The scale's total score can range between 0 and 27 with a higher score indicating greater depressive severity [29, 30]. The PHQ-9 is used by clinicians to monitor depression treatment over time.

### Sociodemographic characteristics

Age, gender, level of education, and marital status, were measured via self-report.

### Household wealth

A wealth index was calculated for each household consisting of assets and living environment characteristics, including rural vs. urban living status, by applying to each component variable its estimated contribution to the 2010 Tanzania Demographic and Health Survey (DHS) wealth index [31]. A linear regression model predicted DHS wealth scores as a function of indicator variables for the presence of electricity, radio, television, motorbike, car, clock/watch, refrigerator, mobile phone, and/or non-mobile phone in the household; pipe-borne drinking water on the property, variables describing the toilet facility (flush toilet vs. other), natural vs. rudimentary floor construction, and whether the household is located in a rural or urban area. Parameter estimates were used to predict a wealth score for each CHAT household as a linear combination of the parameters for each covariate.

### Economic difficulties related to health care

We assessed the economic burden of ART, which was previously shown to affect adherence to ART in Tanzania [12]. Participants were asked whether they had to go without necessities such as food, clothes, or housing in order to use the money for health care or medications. To understand the burden of travel to care, participant's current address was used to generate a binary variable that indicates rural residence in contrast to urban residence or mixed area. For 60 participants for whom address was not available at 36 months, records at 42 months were used instead. Participants rarely moved during the study period.

### Adherence to ART medication

Among study participants prescribed ART, adherence was assessed with three self-report questions. First, each participant was asked a single item from the AIDS Clinical Trial Group adherence assessment [32], namely when the last time was that s/he had missed a dose of his/her antiretrovirals, with response options of never, within the past week, 1–2 weeks ago, 3–4 weeks ago, 1–3 months ago, or more than 3 months ago. Next, the participant was asked to show, on a 0–100% visual analog scale, (1) the percentage of their antiretroviral drugs they had taken in the last month and (2) the percentage of their antiretroviral drugs they had missed in the last month. Participants were classified as adherent if they reported not missing doses (1 and 2). Participants with strongly inconsistent answers to the questions on doses taken and doses missed in the last month were excluded (N = 7).

### Variables Not Included

The CHAT study collected information on other variables that have been seen in other studies to predict non-adherence; however, there was too little variation to be included in these analyses.

#### Pill Burden

Most participants took one or two pills per day and there was not a statistically significant difference in pill burden. In absolute numbers, those who reported perfect adherence had a higher pill burden than those who reported non-adherence. For example, 58% of those reporting perfect adherence took 1 or 2 pills per day relative to 54% of those reporting non-adherence. Eleven percent of perfect adherers took 5 or more pills per day compared to 6% of non-adherers.

#### Negative Side Effects

Only 6 participants reported missing medication doses due to negative side effects from medications, precluding the use of the variable as a predictor.

#### Alcohol Abuse

Reported alcohol use was quite low in this HIV+ population on treatment. Only 2% (1% of the adherence group, 10% of the non-adherent group) reported drinking more than once per week. The mean AUDIT score was 0.5 on a scale of 0–10 (0.4 for adherent group, 1.1 for non-adherent group).

### Statistical Analysis

Means or proportions were compared between established and newly diagnosed HIV patients and between those with complete and incomplete adherence using the Student's *t* test or the chi-square test respectively. A multivariate Poisson regression model was constructed using potential predictors of adherence, identified a priori based on those characteristics demonstrated to be predictive of medication adherence in previous research and based on the study hypotheses. With the binary outcome variable, exponentiated coefficients from the Poisson model are interpretable as risk ratios [33]. Log-binomial models were tested, but did not converge due to small numbers. In addition to demographic and socioeconomic characteristics (age, gender, marital status, education, household wealth, sacrifice of other necessities for healthcare, and rural vs. urban residence), the multivariate regression model included the experience of traumatic events in childhood and separately in adulthood, recent stressful life events, post-traumatic stress symptomatology, depression, social support, time since ART initiation, and a binary variable indicating established vs. newly diagnosed HIV patients. For multivariate regression, we rescaled age so that risk ratios reflected the predicted differences between 5 year age increments. Due to small numbers, childhood traumatic experiences were grouped together in the multivariate model as were adult experiences. The multivariate model was run with and without PTSD as a variable in order to tease out the effect of childhood trauma as mediated by PTSD.

Statistical analyses were conducted using Stata software, version 11.2 (StataCorp).

## Results

Of 547 established and newly diagnosed patients who reported valid answers regarding all covariates used in this analysis, 468 (85.6%) were prescribed ART (Table 1). Approximately three-quarters (78%) were prescribed ART regimens considered first-line therapy according to the 2009 Tanzanian treatment guidelines with once or twice daily dosing [34]. Among those on ART, 82.7% reported complete adherence to ART regimen, and 17.3% reported incomplete adherence. There were no statistically significant differences in demographic and economic characteristics between those adherent and non-adherent to medications: the average age was 41.8; males comprised 29.5% of the population; 38.9% of participants were married or cohabitating, 30.3% were widowed, and 19.0% divorced or separated; 5.8% of participants reported no formal education, 77.1% received only primary school education, and 17.1% had attended secondary school or higher; 53.0% of participants resided in rural area; and average household wealth estimated 9.8 on a scale from to −7.2 to 30, with a higher score indicating a wealthier household.

**Table 1:** Sociodemographic Sample Description.

		HIV + Sample on HIV Medications		
	Total	Adherent	Non-Adherent	p-value
	N = 468	N = 387	N = 81	
Age, Mean (SD)	41.8 (8.4)	42.0 (8.4)	40.7 (8.3)	0.215
Male gender	29.49%	29.72%	28.40%	0.813
Marital status				
Married/cohabitating	38.89%	39.53%	35.80%	0.532
Never married	11.75%	11.63%	12.35%	0.856
Widowed	30.34%	29.97%	32.10%	0.706
Divorced/separated	19.02%	18.86%	19.75%	0.853
Highest level of education achieved				
None	5.77%	6.20%	3.70%	0.382
Primary school (1–6 years)	77.14%	76.49%	80.25%	0.465
Secondary school or higher	17.09%	17.31%	16.05%	0.784
Sacrifice of other necessities for healthcare	22.44%	24.29%	13.58%	0.036
Rural residence	52.99%	54.52%	45.68%	0.148
DHS wealth index, Mean (SD)	9.8 (9.5)	9.8 (9.5)	9.8 (9.5)	0.952
Time in months since ART initiation, Mean (SD)	52.8 (22.6)	54.7 (22.6)	44.1 (20.7)	0.000
Established (vs. recently diagnosed) HIV patient	77.78%	81.65%	59.26%	0.000

Statistically significant differences were found in the percent reporting that they sacrificed other necessities for health care needs (24.5% vs. 13.6%: p-value <0.05) and the mean time since ART initiation (55 months vs. 44 months: (p-value <0.01) between those reporting medication adherence and those reporting incomplete adherence.

Table 2 reports the distribution of types of traumatic events, post-traumatic stress symptomatology, depressive symptom severity, recent stressful life events, and social support. Those not completely adherent to ART reported a mean of 2.5 types of potentially traumatic events, compared with a mean of 2.1 in the completely adherent group. In particular, those with incomplete adherence to ART were more likely than those who were completely adherent to have experienced sexual abuse prior to puberty (17.5% vs. 6.8%: p-value <0.01) and the death of an immediate family member not due to homicide or suicide (64.2 vs. 51.7%: p-value <0.05). In addition, those who were incompletely adherent had higher post-traumatic stress (p-value <0.01) and depressive symptomatology (p-value <0.01) scores and less social support (p-value <0.01) than those who reported complete adherence. Twenty-five percent of the non-adherent group had PHQ-9 scores of 5 or more (compared to a score of 2 for the adherent group), an indication of “mild depression,” with the highest score for this group being 23.

**Table 2:** Life Events, Mental Health Symptomatology and Social Support.

		HIV+ Sample on HIV Medications		
	Total	Adherent	Non-Adherent	p-value
	N = 468	N = 387	N = 81	
Traumatic events				
Childhood trauma category				
Mean experienced (SD)	2.2 (1.5)	2.1 (1.5)	2.5 (1.4)	0.061
Sexual abuse before puberty	8.66%	6.81%	17.50%	0.002
Physical abuse or assault before 13 years old	3.63%	3.36%	4.94%	0.491
Parental alcoholism or use of drugs	59.62%	60.98%	53.09%	0.189
Parental mental illness, suicide, or suicide attempt	9.83%	9.30%	12.35%	0.404
Parent or caregiver went to prison	9.40%	8.79%	12.35%	0.319
Parental fighting and threats	31.20%	31.52%	29.63%	0.738
Emotional neglect	8.97%	9.04%	8.64%	0.909
Physical neglect	20.30%	19.12%	25.93%	0.167
In orphanage/foster care/reformatory/put up for adoption	1.71%	1.55%	2.47%	0.563
Imprisonment for one week or longer	2.99%	2.84%	3.70%	0.680
Life-threatening illness or injury	6.62%	5.68%	11.11%	0.074
Death of immediate family member not due to human action	53.85%	51.68%	64.20%	0.040
Death of a close person due to other person	1.92%	2.07%	1.23%	0.621
Adulthood trauma category				
Mean experienced (SD)	1.0 (0.9)	1.1 (0.9)	1.0 (0.9)	0.652
Sexual abuse since puberty	13.42%	13.09%	15.00%	0.649
Physical abuse or assault since 13 years old	7.26%	6.98%	8.64%	0.600
Death of a close person due to other person	14.32%	14.73%	12.35%	0.579
Death of a child	33.12%	34.11%	28.40%	0.321
Death of spouse or partner	36.32%	36.43%	35.80%	0.915
Lifetime trauma category	3.3 (2.0)	3.3 (2.0)	3.5 (1.8)	0.405
Incident stress category, Mean experienced (SD)	2.3 (2.1)	2.3 (2.1)	2.3 (2.1)	0.824
Mental Health Symptomatology				
PTSD symptomology (Scale range: 0–68), Mean (SD)	11.2 (9.0)	10.5 (8.0)	14.9 (11.9)	0.000
PHQ-9 depressive severity (Scale range: 0–27), Mean (SD)	2.0 (2.9)	1.8 (2.6)	3.3 (4.1)	0.000
Social Support (Scale range: 0–76), Mean (SD)	57.5 (18.8)	58.6 (18.8)	52.3 (18.3)	0.006

Note: Incident stress category excludes (1) those overlapping with adulthood trauma and (2) income or employment-related stress.

### Multivariate Analysis

In a multivariate Poisson regression model (see Table 3) that controlled for each participant's gender, age, marital status, education, household wealth, rural vs. urban residence, status as an established vs. newly diagnosed HIV patient, time since ART initiation, number of types of adulthood traumatic experiences, number of recent stressful life events, PTSD symptomology, and social support, we found that incomplete ART adherence was statistically significantly positively associated with the experience of childhood traumatic events and PHQ-9 depressive symptom severity. Incomplete adherence showed a trend toward negative association with sacrifice of other necessities for health care needs (meaning those with complete adherence were more likely to report making such sacrifices).

**Table 3:** Multivariate Poisson regression of incomplete ART adherence in 468 Tanzanian Adults.

	Incomplete Adherence without PTSD			Incomplete Adherence with PTSD		
Independent Variables	IRR	95% CI	p value	IRR	95% CI	p value
Childhood trauma category (per additional category)	1.15	1.03, 1.28	0.010	1.13	1.02, 1.26	0.026
Adulthood trauma category (per additional category)	0.86	0.66, 1.11	0.247	0.86	0.67, 1.11	0.256
Time since ART initiation	0.99	0.98, 1.00	0.030	0.99	0.98, 1.00	0.064
PHQ-9 depressive severity (per additional unit)	1.12	1.08, 1.17	0.000	1.09	1.04, 1.15	0.001
Incident stress category (per additional category)	0.96	0.86, 1.08	0.518	0.96	0.86, 1.08	0.487
PTSD symptomology (per additional unit)	N/A	N/A	N/A	1.02	1.00, 1.04	0.107
Male gender	1.02	0.63, 1.63	0.940	1.05	0.65, 1.68	0.848
Age in 5 years	0.97	0.86, 1.10	0.663	0.97	0.86, 1.10	0.642
Marital status (ref = married/cohabitating)						
Never married	0.88	0.46, 1.67	0.688	0.87	0.45, 1.67	0.676
Widowed	1.23	0.72, 2.10	0.447	1.16	0.67, 2.01	0.605
Divorced/separated	0.98	0.56, 1.70	0.942	0.92	0.52, 1.62	0.763
Highest education level achieved (ref = primary school)						
None	0.40	0.15, 1.05	0.064	0.45	0.18, 1.12	0.087
Secondary school or more	1.00	0.61, 1.64	0.986	1.00	0.61, 1.63	0.996
Sacrifice of other necessities for healthcare	0.51	0.26, 1.03	0.061	0.52	0.26, 1.03	0.062
DHS wealth index (per additional unit)	1.01	0.98, 1.03	0.647	1.01	0.98, 1.03	0.554
Rural (vs. urban/mixed area) residence	0.72	0.49, 1.06	0.098	0.71	0.48, 1.06	0.091
Established (vs. newly diagnosed) HIV patient	0.67	0.42, 1.07	0.094	0.62	0.38, 1.02	0.059
Social support (per additional unit)	0.99	0.98, 1.00	0.159	0.99	0.98, 1.00	0.201

Each additional childhood traumatic experience was associated with a 13% increase in the risk of incomplete ART adherence (risk ratio  = 1.13; p-value <0.05). Each additional 1-unit increase on the 27-point PHQ-9 depressive symptom severity score was associated with a 9% increase in the risk of incomplete ART adherence (risk ratio  = 1.09; p-value <0.01). Conversely, sacrifice of other necessities for health care was associated with a 48% decrease in the risk of incomplete ART adherence (risk ratio  = 0.52; p-value  = 0.06), suggesting a protective association between such financial sacrifice and ART adherence.

When the model excluded PTSD, the risk ratio for childhood trauma changed only marginally, indicating that PTSD mediated little or none of the relationship between childhood trauma and incomplete adherence. These results suggest that childhood trauma influences incomplete adherence independently of PTSD symptomatology.

Alternate regression models alternatively included binary variables for: childhood sexual abuse, an immediate family member of participant's died not due to human action during participant's childhood, and a refined continuous variable that indicates the number of types of traumas participant experienced among the other types of childhood trauma. The direction and scale of coefficients were intuitively right but due to the small sample size, two of three did not have enough statistical power to show significance. Childhood sexual abuse was positively associated with incomplete adherence to ART with a trend towards statistical significance (RR  = 1.57, p<0.1), an immediate family member of participant's died not due to homicide or suicide during participant's childhood was statistically significantly positively associated with incomplete adherence to ART (RR  = 1.50, p<0.05), and the number of other types of trauma participant experienced during childhood was positively associated with incomplete adherence to ART (RR  = 1.05) but was not statistical significant.

## Discussion

This study indicates that mental health symptomatology and the sequelae of past trauma are associated with non-HIV medication adherence: conditions which could be treated in conjunction with HIV. With the estimated $16.8 billion being spent globally on HIV in 2011 and a goal of increasing spending to $22 to $24 billion by 2015 [35] there is growing recognition that we need to focus not just on availability of medications, but also on adherence to medications and retention in care [3]. This study indicates that expanding HIV services to include mental health services would result in higher levels of medication adherence. Relatively inexpensive and manualized mental health treatment protocols for depression and anxiety are being developed and tested in low income countries and could be feasibly integrated with HIV care.

People non-adherent to medications were on HIV medications for a shorter period of time and this variable was significant in one of the two multivariable models. The reason for this difference may be because people who have been on treatment and engaged in care for a longer period of time are more likely have gained a trusting relationship with providers, or, as others have hypothesized, because those who were the first to have access to ART in low income countries were those who were most motivated to adhere to the medications and were seeking the medications. As medications become more widely available, non-adherence may increase.

Those individuals with incomplete adherence had greater depressive symptoms as measured by the PHQ-9. Similar to findings from our studies in the U.S., people reporting incomplete adherence endorsed prior exposure to more types of childhood potentially traumatic experiences, notably childhood sexual abuse [22]. In multivariable models, childhood trauma and depressive symptom severity were highly significant predictors of incomplete ART adherence.

In interpreting the results of this study, we note several limitations. Many of the measured variables, including ART adherence and exposure to traumatic events symptoms, were measured by self-report. These reports may have been subject to recall bias since most traumatic events occurred years prior to the study interviews. Further, traumatic events may have been under-reported due to reluctance to mention difficult experiences. Conservative cut-off points were used in defining ART adherence. The observational design of the study limits the causal interpretation of these results.

Limitations notwithstanding, it is impressive that mental health symptomatology and trauma sequelea are more predictive of HIV medication adherence than the sociodemographic and economic barriers. While HIV related services are still being created in LMICs, we have a window of opportunity to integrate HIV services with mental health related services. Our findings suggest that exposure to potentially traumatic events and severity of depressive symptoms negatively affect ART adherence in a sub-Saharan African population. There are effective intervention strategies in the U.S. that improve outcomes in HIV+ individuals who have been exposed to traumatic events [36], [37], [38]. Further, treatments for depression, including psychotherapeutic and pharmacologic interventions, have proven effective in improving ART adherence in a number of studies in the US and Switzerland. Small studies of psychotherapeutic interventions in similarly resource-constrained sub-Saharan African settings have demonstrated that interventions for depression are acceptable and effective [39], [40], [41], [42]. As treatment interventions for HIV continue to be rolled out in sub-Saharan Africa, and as the burden of mental illness is increasingly recognized both in its own right and as it relates to prognosis and management of other illnesses, it is essential to understand barriers to care and to identify potential areas for intervention that would facilitate more effective HIV care for millions of people. Allocation of funding in HIV programs such as PEPFAR and the Global Fund to integrate evidence-based interventions to reduce the impact of psychosocial stressors, in particular trauma-related stress, has the potential to greatly impact ART adherence and overall health in HIV-infected individuals.
